# Optimization of Steroid Photochemistry and Its Application in the Synthesis of 5,6‐Dihydro‐Ophiopogonol A

**DOI:** 10.1002/chem.202500395

**Published:** 2025-04-21

**Authors:** Chiara Maioli, Gianluigi Lauro, Anna Sategna, Diego Caprioglio, Hawraz Ibrahim M. Amin, Maurizio D'Auria, Daniela Imperio, Giuseppe Bifulco, Alberto Minassi

**Affiliations:** ^1^ Department of Pharmaceutical sciences Università del Piemonte Orientale Novara Italy; ^2^ Department of Pharmacy Università degli Studi di Salerno Fisciano Italy; ^3^ Department of Chemistry Università degli Studi di Pavia Pavia Italy; ^4^ Department of Sciences Università degli Studi della Basilicata Potenza Italy; ^5^ Department for Sustainable Development and Ecological Transition Università del Piemonte Orientale Vercelli Italy

**Keywords:** natural products, photochemistry, spirostane, steroids, structure elucidation

## Abstract

The photoreactivity of steroids represented a hot topic in the middle of the last century and in this project, we “rediscover” it through the exploration of the photochemical behavior of Δ^1^‐3‐keto‐steroids. In terms of number of products obtained, the photochemistry of Δ^1^‐3‐keto‐steroids is less complicated than that of Δ^4^‐3‐keto‐ and Δ^1,4^‐3‐keto‐steroids, furnishing an efficient and tunable method to remodel the classic steroid 6/6/6/5 ring system. In this scenario, this approach can represent a simple strategy to interconvert a class of easily available steroids to another difficult‐to‐access from natural sources. As a proof of concept, the synthesis 5,6‐dihydro‐ophiopogonol A (**11**), a very close analog of natural ophiopogonol A (**7**), was accomplished in just four steps starting from easily available diosgenin (**8**).

## Graphical abstract

Steroid photoreactivity was a major topic in the last century. This study revisits it by exploring the photochemistry of Δ^1^‐keto‐steroids, offering an efficient method to modify the classic steroid 6/6/6/5 ring system. This approach enables the transformation of accessible steroids into rare ones, demonstrated by synthesizing 5,6‐dihydro‐ophiopogonol A in four steps from diosgenin.

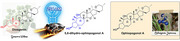

## 1 Introduction

The term “steroids” was proposed for the first time in 1936 by Callow and Young to classify “the group of biologically important compounds containing the reduced cyclopentanophenanthrene ring system,” comprising the sterols, cardiac aglycones, bile acids, and sex hormones.^[^ ^1^ ^]^ Since their identification, steroids have played an important role in the drug discovery, thanks to their excellent ability to penetrate cell membranes, and bind to nuclear and membrane receptors.^[^ ^2^, ^3^ ^]^ The study of their reactivity and the need to synthesize new, more powerful, and selective derivatives have proved to be a spectacular training ground for the progress of organic chemistry. In particular, the combination of photochemistry and steroids has been very fruitful leading to the discovery of new reactions, among which Barton nitrite ester photolysis stands out as the first example of activation of the C─H bond.^[^ ^4^ ^]^

The exploration of the photoreactivity of testosterone‐like compounds represented a hot topic in the middle of the last century due to their fascinatingly complex photochemical behavior. The photolabile nature of the cyclohexenone system could undergo different rearrangements furnishing compounds that are characterized by unique structures unattainable by the more classic organic pathways. The irradiation in neutral media affords a highly strained product resulting from the *lumi*‐ketone rearrangement, while in mild acidic media a mixture of photoproducts is obtained.^[^ ^5^, ^6^, ^7^, ^8^, ^9^ ^]^ More recently, there has been renewed attention to the photochemistry of steroids thanks to the work of Albini's group, who reported interesting results about the photoreactivity and the phototoxic behavior of a set of pregna‐1,4‐dien‐3,20‐diones derivatives.^[^ ^10^, ^11^ ^]^ Despite the great interest and the high number of papers on this topic, surprisingly, most of the studies on natural compounds were limited to the photoreactivity of Δ^4^‐3‐keto‐ and Δ^1,4^‐3‐keto‐steroids neglecting the Δ^1^‐3‐keto‐steroids with just limited examples and confusing information about their photochemical behavior.^[^ ^12^, ^13^ ^]^ This oversight could preclude on one side the discovery of new photorearrangements, and on the other side the access to a new class of steroidal architectures with interesting pharmacological profiles.

To fill the gap and to add a new tile to the photochemical scenario of testosterone‐like compounds, in this study is described the photoreactivity of cholest‐1‐en‐3‐one (**1**),^[^ ^14^ ^]^ as an easily accessible representative of this class of steroids.

## 2 Results and Discussion

**Scheme 1:**
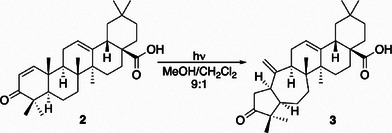
Photochemistry of Δ^1^‐3‐oxo‐oleanolic acid.

Recently it has been demonstrated for the Δ^1^‐3‐oxo‐oleanolic acid (**2**) that the presence of only one double bond at C1 inhibits the classic *lumi*‐ketone rearrangement in favor of the reorganization of the A/B system to get access to new 5(10→1)*abeo*‐oleanolic acid (**3**) (Scheme 1).^[^ ^15^ ^]^

Although cholest‐1‐en‐3‐one (**1**) and Δ^1^‐3‐oxo‐oleanolic acid (**2**) belong to different classes of secondary metabolites, they maintain a high structural and stereochemical homology in the A/B ring system having as main differences a methylene group in place of a *gem*‐dimethyl element at C4 and a hydrogen for a methyl group at C8, respectively. Given their structural homology, the same putative photoreactivity profile was investigated, so compound **1** was irradiated at 254 nm (5.5 W, pen‐ray UV lamp) in degassed neutral medium (MeOH/CH_2_Cl_2_ 9:1) until the disappearance of the starting material, obtaining compounds **4** and **5** in 14% and 43% yield respectively. In light of the role played by the solvent in the formation of the products, compound **1** was irradiated in non‐nucleophilic solvents with different degrees of polarity. The reactions were performed in toluene, dichloromethane, acetone and acetonitrile. While in toluene cholest‐1‐en‐3‐one (**1**) was stable after 60 h of irradiation, performing the reaction in acetone led to a complete degradation of the starting material. Otherwise, by running the reaction in dichloromethane or acetonitrile, neither compounds **4** nor **5** were formed, but 5(10→1)*abeo*‐colesten‐3‐one **6** was obtained in 27% and 37% yield respectively (Table 1).

**Table 1:** Photochemical products and solvent screening.

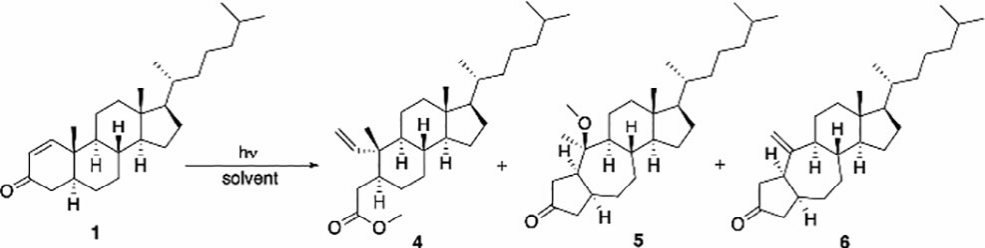				
Solvent	1	4	5	6
MeOH/DCM 9:1	‐	14%^a^	43%^a^	‐
Toluene	100%	‐	‐	‐
DCM	‐	‐	‐	27%a
ACN	‐	‐	‐	37%^a^
Acetone	Degraded	‐	‐	‐

^a^ Isolated yields.

The chemical structures of **4**–**6** were confirmed through the analysis of HRMS data, 1D‐NMR (^1^H and ^13^C) and 2D‐NMR (COSY, HSQC, HSQC‐TOCSY, HBMC) spectra (Tables S1 and S2). Furthermore, the QM/NMR combined approach was applied to shed light about the absolute configuration of **4** and **5**.^[^ ^16^ ^]^ In details, the structural study of these compounds involved the determination of the configuration at the C‐10 since the configurations at the other stereocenters had previously been determined as 5*S*,8*S*,9*S*,13*R*,14*S*,17*R*,20*R* for compound **4** and as 1*R*,5*S*,8*S*,9*S*,13*R*,17*R*,20*R* for **5** (Table 1). For **4** and **5**, the two possible stereoisomers differing for the configuration at C‐10 were then considered (i.e., **4a**/**4b** and **5a**/**5b**, respectively, see Chart S1 and Chart S2, Supporting Information) for the calculation at the density functional theory (DFT) level of ^1^H and ^13^C chemical shift values, which were subsequently compared with the related experimental values. In this way, the configuration at C‐10 was determined as *S* for both **4** and **5** through the analysis of the mean absolute error (MAE) values and DP4+ probabilities^[^ ^17^ ^]^ (Tables S4, S5, and S8, Supporting Information).

**Scheme 2:**
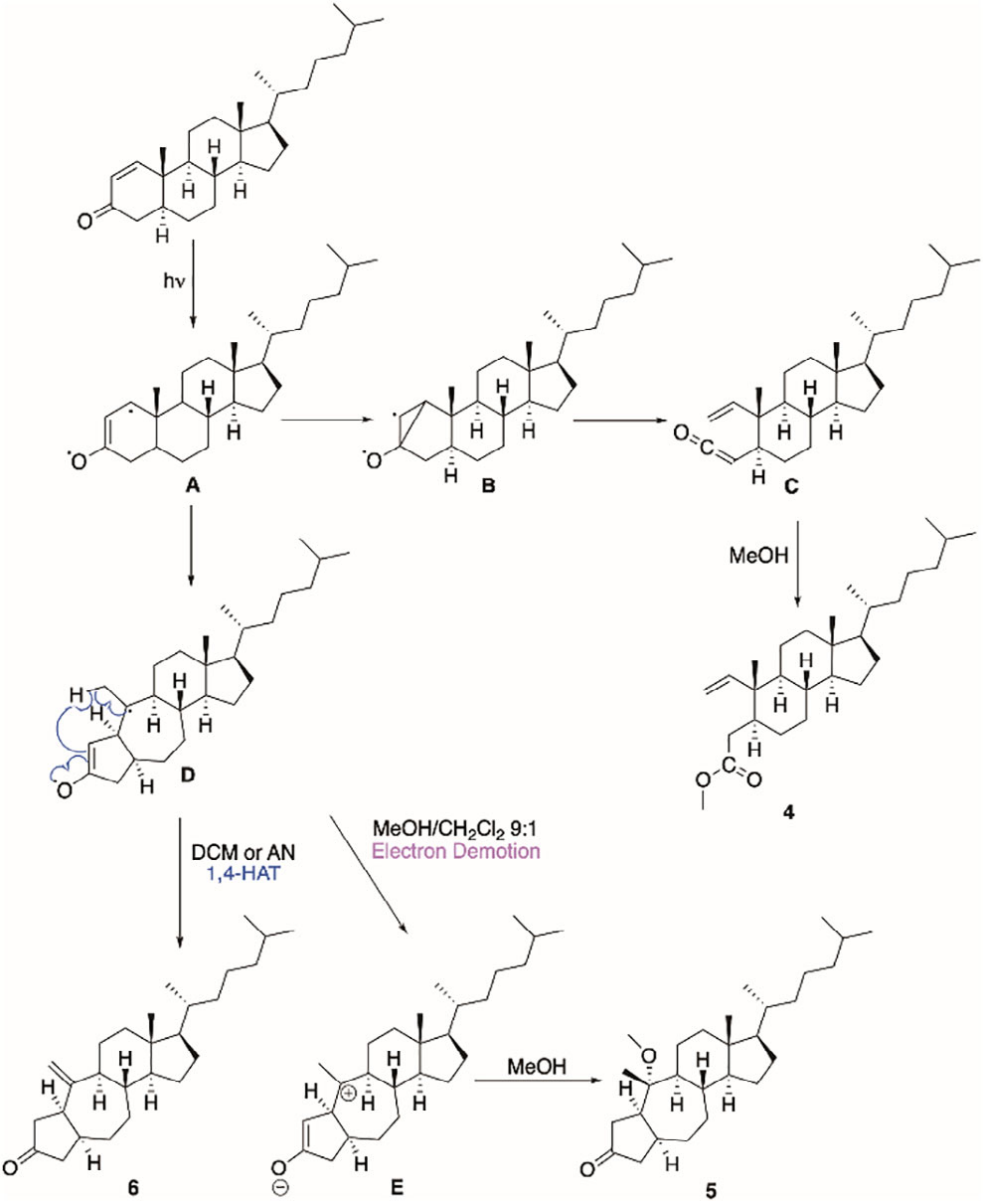
Proposed mechanism for the synthesis of compounds **4**, **5**, and **6**.

Given the compounds resulted from the irradiation, a possible mechanism was proposed. Compound **4** could derive from the activation of the enone moiety leading to diradical **A** that can give intermediate **B** through the formation of a new C─C bond between C1 and C3. The latter can rearrange to ketene **C** eventually trapped by the solvent leading to the desired compound. This reaction cannot occur in aprotic solvent because the formation of **B** is not thermodynamically favored. DFT calculations at B3LYP/6‐311G++(d,p) level of theory showed that **B** was less stable than **A** for 24 kcal mol^−1^. The reaction occurred and gave a product only when the protic solvent allowed to shift the equilibrium between **A** and **B**. On the other hand, compounds **5** and **6** could share the same mechanism starting from diradical **A** that undergoes to a concerted bond rearrangement with the formation of intermediate **D** through the cleavage of the C5–C10 bond and the formation of a new C1–C5 bond with the 5/7 ring system installed. At this point, intermediate **D**, depending on the solvent of the reaction, can follow two different pathways: in MeOH/DCM it could be subjected to an electron‐demotion, as postulated by Zimmerman for other cyclohexenones,^[^ ^18^, ^19^, ^20^, ^21^ ^]^ to form zwitterion **E** with a tertiary carbocation that is stereoselectively trapped by the solvent to give compound **5**. Otherwise, in DCM or ACN, it could directly undergo a 1,4‐HAT (Hydrogen Atom Transfer) giving compound **6** (Scheme 2).^[^ ^22^ ^]^

Spirostanols are a class of steroids characterized by a typical 6/6/6/5/5/6 ring system produced both by plants and animals, that have shown interesting pharmacological activities, such as anticancer, antibacterial and anti‐inflammatory.^[^ ^23^, ^24^, ^25^, ^26^ ^]^ Ophiopogonol A (**7**) is a spirostanol characterized by an unprecedent 5/7/6/5/5/6 ring system, that has been recently isolated from *Ophiopogon japonicus*, a plant whose rhizome is used in the Chinese Traditional Medicine for the treatment and the prevention of lung diseases.^[^ ^27^ ^]^ Ophiopogonol A (**7**), such as the other members of this group of secondary metabolites, can be obtained only in very low amounts (10 mg from 17.5 kg of plant material), a highly limiting factor in the exploration of its pharmacological profile.^[^ ^27^, ^28^ ^]^

Considering the results obtained with compound **1**, the previously described approach may be considered as a simple strategy to interconvert a class of easily available steroids to another difficult‐to‐access from natural sources. To test this hypothesis, the commercially available spirostanol diosgenin (**8**), characterized by a common 6/6/6/5/5/6 system, was chosen as possible precursor to get access to unnatural analogs of compound **7** with an uncommon 5/7/6/5/5/6 architecture. Diosgenin (**8**) has been manipulated to obtain the corresponding Δ^1^‐3‐oxo‐derivative (**9**) that has been transformed into the rearranged spirostanol analog **10** by using a one‐pot operation (irradiation in MeOH/DCM 9:1, then NaBH_4_) with an overall yield over two steps of 30%.

**Scheme 3:**
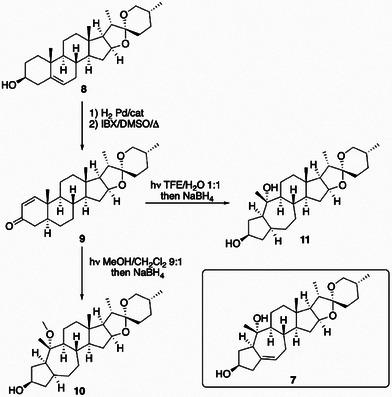
Synthesis of compounds **10** and **11** starting from diosgenin (**8**).

Compound **10** was completely characterized through the analysis of HRMS and 1D and 2D NMR spectra. Also in this case, the QM/NMR approach was employed to determine the absolute configuration, considering in this case the four possible stereoisomers differing for the configurations at the C‐3 and C‐10 stereocenters (**10a**–**10d**, see Chart S3, Supporting Information). The comparison of the experimental ^1^H/^13^C chemical shifts and those predicted at the DFT level highlighted the lowest ^1^H/^13^C MAE values and the best DP4+ probability for **10b**, thus disclosing 1*R*,3*R*,5*S*,8*S*,9*S*,10*S*,13*R*,17*R*,20*R* absolute configuration (Scheme 3 and Tables S6 and S8, Supporting Information).

Having tested the strategy, and trying to get closer to ophiopogonol A (**7**) by replacing the methoxy group at C10 with a hydroxyl one, compound **9** was irradiated in a degassed binary mixture of trifluoroethanol (TFE):water 1:1. TFE is transparent to UV light, it is not nucleophilic but it is necessary to bring our starting material in solution. On the other side, water is the nucleophile for carbocation trapping, preventing any rearrangement and the formation of the double bond such as in compound **6**. Eventually compound **9** dissolved in TFE/water 1:1 was irradiated until the disappearance of the starting material. The reaction was worked‐up and the crude dissolved in MeOH in presence NaBH_4_ to give the desired 5,6‐dihydro‐ophiopogonol A (**11**) in 20% yield (Scheme 3). Again, after characterizing **11** with HRMS and 1D‐ and 2D‐NMR experiments, the experimental and predicted ^1^H/^13^C chemical shifts for the four possible stereoisomeric solutions (**11a**–**11d**) were compared, allowing to confirm the absolute configuration as 1*R*,3*R*,5*S*,8*S*, 9*S*,10*S*,13*R*,17*R*,20*R* (Scheme 3 and Tables S7 and S8).

## 3 Conclusion

It can be concluded that the photochemistry of steroids has been renewed by adding a new tile to the scenario. The photoreactivity of Δ^1^‐3‐keto‐steroids is less complicated, in terms of number of products obtained, compared to that of Δ^4^‐3‐keto‐ and Δ^1,4^‐3‐keto‐steroids and it can be easily tuned by the solvent used. In particular, a new *seco*‐derivative of cholesterol (**4**) was described through an unprecedent cleavage of the C2–C3 bond. On the other hand, the classic steroid 6/6/6/5 ring system was remodeled to a less common 5/7/6/5, and this strategy was used to interconvert an easily available steroid into rarer one as demonstrated with the synthesis of compound **10**. Furthermore, as a proof of concept, 5,6‐dihydro‐ophiopogonol A (**11**), a very closed analog of natural ophiopogonol A (**7**), was obtained in just four steps starting from commercially available diosgenin (**8**). This approach could represent a new semisynthetic strategy to get access to unknown 5(10→1)*abeo*‐steroids with possible new bioactivities.

## 4 Experimental Section

### General experimental procedures

Optical rotations were measured on an Anton Paar Polarimeter MCP 100 at 20°C. Infrared spectra were recorded on a FT‐IR Bruker Alpha II spectrometer with absorption maxima (νmax) recorded in wavenumbers (cm^−1^).

NMR spectra of compounds **4**, **5**, **6**, **10** were recorded in CDCl_3_ (99.8%, Sigma‐Aldrich) using a Bruker DRX‐600 MHz spectrometer (Bruker BioSpin GmBH, Rheinstetten, Germany) equipped with 5 mm PATXI 1H/D‐13C/15N Z‐GRD Z816801/0193 probe and an autosampler at *T* = 298.0 K at 600.17 MHz (^1^H) and 150 MHz (^13^C). Standard pulse sequences and phase cycling were utilized for COSY, HSQC‐EDITED, HSQC‐TOCSY, and HMBC spectra. Chemical shifts are reported in parts per million (ppm) relative to the residual solvent. The NMR spectra of compounds **9** and **11** were recorded using a Bruker Avance Neo 400 MHz spectrometer (Bruker BioSpin GmBH, Rheinstetten, Germany) in CDCl_3_ (99.8%, Sigma‐Aldrich) equipped with Z163739_0095 (PI HR‐400‐S1‐BBF/H/D‐5.0‐Z SP) probe at *T* = 298.0 K at 400 MHz (^1^H) and 100 MHz (^13^C). Data processing for NMR experiments was carried out with Topspin 3.2 software.

A Q‐Exactive Plus UHMR Hybrid Quadrupole Orbitrap Mass Spectrometer (Waltham, MA, USA) was employed for mass spectrometry. The spectra were recorded by infusion into the ESI source using MeOH as the solvent. Commercially available reagents and solvents were purchased from Aldrich, TCI and Fluorochem and were used without further purification. Column chromatography was performed on silica gel (Merck Kieselgel 60, 230−400 mesh ASTM). Thin‐layer chromatography (TLC) was carried out on 5 × 20 cm plates with a layer thickness of 0.25 mm (Merck silica gel 60 F254). When necessary, KMnO_4_ was used for visualization. Compound **1** has previously been reported.^[^ ^14^ ^]^

**Synthesis of (2aS,5′R,6aR,6bS,8aS,8bR,9S,10R,11aS,12aS,12bS)‐5′,6a,8a,9‐tetramethyl‐1,2a,3,3′,4′,5′,6a,6b,6′,7,8,8a,8b,9,11a,12,12a,12b‐octadecahydrospiro[naphtho[2′,1′:4,5] indeno [2,1‐b]furan‐10,2′‐pyran]‐4(2H)‐one (9)**: A solution of diosgenin (8) (1 gr, 2.41 mmol) and a catalytical quantity of Pd/C 10% in MeOH/EtOAc 9:1 was stirred under a H_2_ atmosphere (baloon) overnight. The mixture was filtered over a celite pad, and the organic phases were evaporated under vacuum. The crude was dissolved in benzene/DMSO (4:1) (30 mL) and IBX (2‐iodoxybenzoic acid) (3.37 g, 12.05 mmol, 5 mol/equivalent) and a catalytic amount of fluorobenzene were sequentially added. The reaction was heated at 100°C overnight, quenched with Na_2_SO_3_ (saturated solution), and extracted with EtOAc. The organic phases were washed with NaHCO_3_ (saturated solution), brine, dried over Na_2_SO_4_ and evaporated. The crude was purified over silica gel (petroleum ether/EtOAc, 9:1), affording 490 mg (50%) as a white powder. ^1^H‐NMR (400 MHz, CDCl_3_) δ 7.15 (d, *J* = 10.2 Hz, 1H), 5.87 (dd, *J* = 10.1, 1.0 Hz, 1H), 4.42 (ddd, *J* = 8.6, 7.6, 6.2 Hz, 1H), 3.49 (ddd, *J* = 10.9, 4.7, 2.0 Hz, 1H), 3.39 (t, *J* = 10.9 Hz, 1H), 2.39 (dd, *J* = 17.7, 14.1 Hz, 1H), 2.24 (ddd, *J* = 17.7, 4.1, 1.0 Hz, 1H), 1.04 (s, 3H), 0.99 (d, *J* = 6.9 Hz, 3H), 0.83 (s, 3H), 0.81 (d, *J* = 6.4 Hz, 3H); ^13^C‐NMR (100 MHz, CDCl_3_) δ 200.2, 158.3, 127.4, 109.3, 80.7, 66.9, 62.2, 56.2, 50.0, 44.3, 41.6, 41.0, 40.7, 39.8, 39.1, 35.3, 31.7, 31.5, 31.4, 30.3, 28.8, 27.6, 21.1, 17.1, 16.6, 14.5, 13.1.

### General photochemical procedure

A degassed solution of 100 mg of Δ^1^‐3‐enone (1 eq/mol.) in 15 mL of solvent (MeOH/CH_2_Cl_2_ 9:1; toluene; DCM; AN; acetone; TFE/H_2_O 1:1) was irradiated (5.5 W, high pressure mercury lamp) until the disappearance of the starting material checked by TLC. The solvent was evaporated at reduced pressure and the crude was purified over silica gel (compounds **4**, **5**, **6**) or reduced with NaBH_4_ in MeOH and purified over silica gel (compounds **10**, **11**).

**Methyl 2‐((3R,3aR,5aS,6S,7S,9aS,9bS)‐3a,6‐dimethyl‐3‐((R)‐6‐methylheptan‐2‐yl)‐6‐vinyldodecahydro‐1H‐cyclopenta[a]naphthalen‐7‐yl)acetate (4)**: [a]^20^_D_ +1.99 (c 0.5 acetone); IR (KBr) 2929, 2867, 2849, 1738, 1435, 1160, 910; HRESIMS m/z 439.35455 [M+Na]^+^ (calcd for C_28_H_48_NaO_2_, 439.35465).

^1^H‐NMR (600.17 MHz, CDCl_3_) δ 5.51 (dd, *J* = 17.5, 10.8 Hz, 1H), 5.06 (d, *J* = 10.8 Hz, 1H), 4.88 (d, *J* = 17.5 Hz, 1H), 3.62 (s, 3H), 2.39 (dd, *J* = 15.2, 2.5 Hz, 1H), 1.90 (m, 1H), 1.88 (m, 1H), 1.82 (m, 1H), 1.70 (m, 1H), 1.66 (m, 1H), 1.61 (m, 1H), 1.55 (m, 1H), 1.50 (n, *J* = 6.6 Hz, 1H), 1.35 (m, 1H), 1.33 (m, 1H), 1.31 (m, 2H), 1.27 (m, 1H), 1.24 (m, 1H), 1.23 (m, 1H), 1.22 (m, 1H), 1.12 (m, 2H), 1.10 (m, 1H), 1.08 (m, 1H), 1.06 (m, 1H), 1.04 (m, 1H), 0.99 (m, 1H), 0.98 (m, 1H), 0.92 (dd, *J* = 12.5, 3.4 Hz, 1H), 0.87 (d, *J* = 6.5 Hz, 3H), 0.85 (d, *J* = 6.4 Hz, 3H), 0.84 (d, *J* = 6.4 Hz, 3H), 0.84 (m, 1H), 0.82 (s, 3H), 0.63 (s, 3H); ^13^C‐NMR (150 MHz, CDCl_3_) δ 174.7, 148.3, 113.3, 56.7, 56.3, 51.5, 43.4, 42.8, 40.1, 39.7, 36.6, 36.3, 35.9, 35.1, 31.5, 28.4, 28.1, 27.4, 24.2, 24.0, 23.0, 22.7, 18.8, 12.2, 10.4.

**(3R,3aR,5aS,6S,6aR,9aS,11aS,11bS)‐6‐methoxy‐3a,6‐dimethyl‐3‐((R)‐6‐methylheptan‐2‐yl)hexadecahydro‐8H‐indeno[5,4‐f]azulen‐8‐one (5)**: [a]^20^_D_ +39.63 (c 2.5 CHCl_3_); IR (KBr) 2931, 2867, 1731, 1456, 1382, 1168, 1061, 800; HRESIMS m/z 439.35475 [M+Na]^+^ (calcd for C_28_H_48_NaO_2_, 439.35465).

^1^H‐NMR (600.17 MHz, CDCl_3_) δ 3.00 (s, 3H), 2.74 (d, *J* = 19.8 Hz, 1H), 2.73 (t, *J* = 6.7 Hz, 1H), 2.64 (dqd, *J* = 11.0, 8.4, 6.2 Hz, 1H), 2.35 (m, 1H), 2.33 (m, 1H), 2.17 (dt, *J* = 14.5, 8.4 Hz, 1H), 2.00 (m, 1H), 1.99 (bd, *J* = 13.3 Hz, 1H), 1.83 (m, 2H), 1.80 (m, 1H), 1.67 (m, 1H), 1.64 (m, 1H), 1.52 (n, *J* = 6.5 Hz, 1H), 1.44 (m, 1H), 1.38 (m, 1H), 1.34 (m, 1H), 1.33 (m, 2H), 1.27 (m, 1H), 1.26 (m, 2H), 1.17 (m, 1H), 1.15 (m, 1H), 1.14 (m, 1H), 1.13 (m, 2H), 1.11 (m, 1H), 1.00 (m, 1H), 1.00 (s, 3H), 0.99 (qnt, *J* = 4.3 Hz, 1H), 0.92 (d, *J* = 6.4 Hz, 3H), 0.87 (d, *J* = 6.4 Hz, 6H), 0.72 (s, 3H); ^13^C‐NMR (150 MHz, CDCl_3_) δ 220.6, 80.2, 57.1, 56.6, 52.3, 47.6, 45.8, 43.8, 43.2, 41.9, 41.8, 40.2, 39.6, 38.2, 36.2, 35.6, 32.8, 30.2, 28.2, 28.1, 25.5, 23.5, 22.9, 22.7, 19.1, 18.7, 12.4.

**(3R,3aR,5aS,6aR,9aS,11aR,11bS)‐3a‐methyl‐6‐methylene‐3‐((R)‐6‐methylheptan‐2‐yl)hexadecahydro‐8H‐indeno[5,4‐f]azulen‐8‐one (6)**: [a]^20^_D_ +6.94 (c 5 CHCl_3_); IR (KBr) 2930, 2867, 1733, 1465, 1443, 1380, 1264, 1167, 1035; HRESIMS m/z 407.20398 [M+Na]^+^ (calcd for C_27_H_44_NaO, 407.32844).

^1^H‐NMR (600.17 MHz, CDCl_3_) δ 4.99 (s, 1H), 4.61 (s, 1H), 3.02 (q, *J* = 7.8 Hz, 1H), 2.51 (m, 1H), 2.41 (dd, *J* = 19.1, 8.4 Hz, 1H), 2.38 (dd, *J* = 19.1, 5.0 Hz, 1H), 2.02 (m, 1H), 2.00 (dd, *J* = 3.1, 1.6 Hz, 1H), 1.97 (m, 1H), 1.85 (d, *J* = 5.6 Hz, 1H), 1.84 (d, *J* = 4.8 Hz, 1H), 1.83 (m, 1H), 1.78 (m, 2H), 1.77 (m, 1H), 1.66 (dd, *J* = 9.3, 3.6 Hz, 1H), 1.62 (m, 1H), 1.51 (m, 1H), 1.45 (q, *J* = 3.6 Hz, 1H), 1.37 (m, 1H), 1.33 (m, 1H), 1.25 (t, *J* = 11.4 Hz, 1H), 1.23 (m, 1H), 1.20 (t, *J* = 3.8 Hz, 1H), 1.18 (d, *J* = 4.1 Hz, 1H), 1.17 (m, 1H), 1.14 (m, 1H), 1.12 (m, 1H), 1.11 (m, 1H), 1.10 (m, 1H), 1.09 (m, 1H), 1.06 (m, 1H), 0.92 (d, *J* = 6.6 Hz, 3H), 0.86 (d, *J* = 6.4 Hz, 3H), 0.85 (d, *J* = 6.4 Hz, 3H), 0.76 (m, 3H); ^13^C‐NMR (150 MHz, CDCl_3_) δ 220.0, 154.8, 107.9, 57.3, 56.3, 51.6, 46.0, 45.8, 44.9, 44.3, 43.0, 40.1, 38.9, 36.3, 35.9, 32.1, 31.9, 28.3, 28.2, 28.1, 25.8, 24.7, 23.9, 22.9, 22.7, 18.8, 12.4.

**(2R,3S,3aR,3bS,5aS,5′R,6S,6aR,8R,9aS,11aS,11bS,12aS)‐6‐methoxy‐3,3b,5′,6‐tetramethyldocosahydrospiro[azuleno[6′,5′:4,5]indeno[2,1‐b]furan‐2,2′‐pyran]‐8‐ol (10)**: [a]^20^_D_ −34.83 (c 1.4 CHCl_3_); IR (KBr) 3416, 2926, 2908, 2870, 2845, 1450, 1374, 1174, 1050, 980, 898; HRESIMS m/z 469.32833 [M+Na]^+^ (calcd for C_28_H_46_NaO_4_, 469.32833).

^1^H‐NMR (600.17 MHz, CDCl_3_) δ 4.37 (q, *J* = 7.5 Hz, 1H), 4.15 (bqnt, *J* = 8.1 Hz, 1H), 3.46 (bd, *J* = 10.8 Hz, 1H), 3.37 (t, *J* = 10.8 Hz, 1H), 3.02 (s, 3H), 2.42 (q, *J* = 9.1 Hz, 1H), 2.18 (dt, *J* = 13.9, 8.3 Hz, 1H), 2.05 (m, 2H), 1.96 (qnt, *J* = 5.6 Hz, 1H), 1.88 (q, *J* = 6.8 Hz, 1H), 1.84 (m, 1H), 1.77 (m, 1H), 1.76 (m, 2H), 1.71 (dt, *J* = 12.8, 3.5 Hz, 1H), 1.66 (dd, *J* = 9.2, 4.3 Hz, 1H), 1.63 (m, 1H), 1.62 (m, 1H), 1.60 (m, 1H), 1.58 (m, 1H), 1.48 (m, 1H), 1.43 (m, 2H), 1.35 (m, 1H), 1.31 (m, 2H), 1.29 (m, 1H), 1.20 (m, 1H), 1.17 (s, 3H), 1.12 (td, *J* = 12.8, 3.5 Hz, 1H), 0.96 (d, *J* = 6.8 Hz, 3H), 0.85 (q, *J* = 12.0 Hz, 1H), 0.82 (s, 3H), 0.78 (d, *J* = 6.2 Hz, 3H); ^13^C‐NMR (150 MHz, CDCl_3_) δ 109.3, 80.7, 80.5, 72.3, 67.0, 62.5, 57.0, 52.4, 47.9, 44.7, 43.6, 41.9, 41.0, 40.3, 39.4, 37.8, 32.9, 32.7, 31.5, 31.1, 30.4, 28.9, 23.2, 19.3, 17.3, 16.9, 14.7.

**(2R,3S,3aR,3bS,5aS,5′R,6S,6aR,8R,9aS,11aS,11bS,12aS)‐3,3b,5′,6‐tetramethyldocosahydrospiro[azuleno[6′,5′:4,5]indeno[2,1‐b]furan‐2,2′‐pyran]‐6,8‐diol (11)**: [a]^20^_D_ −26.06 (c 0.4 CHCl_3_); IR (KBr) 3388, 3325, 2947, 2925, 2870, 1454, 1259, 1174, 1056, 980, 897, 797; HRESIMS m/z 455.31257 [M+Na]^+^ (calcd for C_27_H_44_NaO_4_, 455.31318).

^1^H‐NMR (400 MHz, CDCl_3_) δ; 4.38 (q, *J* = 7.5 Hz, 1H), 4.14 (m, 1H), 3.47 (m, 1H), 3.36 (t, *J* = 10.9 Hz, 1H), 2.22 (m, 2H), 2.05 (m, 1H), 2.01 (m, 1H), 2.00 (m, 1H), 1.94 (m, 1H), 1.88 (q, *J* = 7.0 Hz, 1H), 1.80 (m, 1H), 1.77 (m, 1H), 1.76 (m, 1H), 1.72 (m, 2H), 1.67 (m, 1H), 1.63 (m, 1H), 1.62 (m, 1H), 1.58 (m, 1H), 1.47 (m, 2H), 1.44 (m, 1H), 1.43 (m, 1H), 1.41 (m, 1H), 1.30 (m, 1H), 1.27 (m, 1H), 1.25 (s, 3H), 1.18 (m, 1H), 1.15 (m, 2H), 0.96 (d, *J* = 7.0 Hz, 3H), 0.81 (s, 3H), 0.78 (d, *J* = 6.2 Hz, 3H); ^13^C‐NMR (100 MHz, CDCl_3_) 109.3, 80.5, 77.2, 72.2, 67.0, 62.4, 57.7, 56.7, 51.6, 43.5, 42.0, 40.8, 40.1, 39.9, 39.5, 38.4, 33.8, 32.9, 31.5, 31.0, 30.4, 29.0, 23.6, 19.9, 17.3, 16.7, 14.7.

## Conflict of Interests

The authors declare no conflicts of interest.

## Supporting information



The authors have reported computational and NMR assignment details within the Supporting Information.

## Data Availability

The data that support the findings of this study are available from the corresponding author upon reasonable request.
