# Supplementary material for: Optimization of Steroid Photochemistry and Its Application in the Synthesis of 5,6‐Dihydro‐Ophiopogonol A
Source: Chemistry. 2025 Apr 21;31(29):e202500395. doi: 10.1002/chem.202500395 (PMC12099188; doi:10.1002/chem.202500395)
Supplement: Supplementary file 1 — The authors have reported computational and NMR assignment details within the Supporting Information. [file CHEM-31-e202500395-s001.docx]

**Supporting Information**

**Optimization of Steroid Photochemistry and Its Application in the Synthesis of 5,6-dihydro-ophiopogonol A.**

Chiara Maioli,^[a]^ Gianluigi Lauro,^[b]^ Anna Sategna,^[a]^ Diego Caprioglio,^[a]^ Hawraz Ibrahim M. Amin,^[c]^ Maurizio D’Auria,^[d]^ Daniela Imperio,^[e]^ Giuseppe Bifulco,* ^[b]^ and Alberto Minassi*^[a]^

[a] C. Maioli, A. Sategna, D. Caprioglio, D. Imperio, A. Minassi
Department of Pharmaceutical sciences,
Università del Piemonte Orientale
28100 Novara, Italy
E-mail: [alberto.minassi@uniupo.it](mailto:alberto.minassi@uniupo.it)

[b] G. Lauro, G. Bifulco
Department of Pharmacy
Università degli Studi di Salerno
84084 Fisciano, Italy

E-mail: [bifulco@unisa.it](mailto:bifulco@unisa.it)

[c] H. I. M. Amin
Department of Chemistry
Università degli Studi di Pavia
27100 Pavia, Italy

[d] M. D’Auria
Department of Sciences
Università degli Studi della Basilicata
85100 Potenza, Italy

[e] D. Imperio

Department for Sustainable Development and Ecological Transition

Università del Piemonte Orientale

13100 Vercelli, Italy

**Computational details.** Maestro (Schrödinger Suite) ^[1]^was used to generate the starting 3D chemical structures of the compounds under investigation. Optimization of the 3D structures was performed with MacroModel (Schrödinger Suite)^[2]^ using the OPLS force field and the conjugate gradient Polak-Ribier (PRCG) algorithm. The 3D structures were used as input to perform exhaustive conformational searches at the MM level. Monte Carlo Molecular Mechanics (MCMM) (50000 steps) and Low Mode Conformational Search (LMCS) (50000 steps) were performed (MacroModel, Schrödinger Suite).^[2]^ Molecular dynamics simulations were also performed at 450, 600, 700 and 750 K, setting a time step of 2.0 fs, with equilibration of 0.1 ns and setting a simulation time of 10 ns (MacroModel, Schrödinger Suite).^[2]^ For each isomer, all the sampled conformers were minimized (PRCG, maximum derivative of less than 0.001 kcal/mol) and compared, using the “Redundant Conformer Elimination” module of MacroModel (Schrödinger Suite),^[2]^ specifically excluding those that differed by more than 21.0 kJ/mol (5.02 kcal/mol) from the most energetically favored conformation and setting a minimum limit of 0.5 Å RMSD (root-mean-square deviation) for saving structures.

All subsequent QM calculations were performed with Gaussian 09 software.^[3]^ Specifically, the sampled conformers were optimized at the DFT level using the MPW1PW91 functional and the 6-31G(d) basis set.^[4]^ Subsequently, the optimized geometries were further visually inspected to remove any new redundant conformers. The ^13^C and ^1^H NMR chemical shifts were predicted for each conformer of the possible isomers of the investigated compounds using the MPW1PW91 functional and the 6-31G(d,p) basis set.^[4]^ For each compound, the final datasets containing the ^13^C and ^1^H NMR chemical shifts for each isomer studied were produced, considering the influence of each conformer on the total Boltzmann distribution and considering their relative energies.

The DP4+ probability^[5]^ values for each isomer were calculated considering the ^13^C chemical shift and ^1^H NMR values, using the tool available online and released by the Sarotti research group (https://sarotti-nmr.weebly.com/).

**Table S1**. NMR data recorded in CDCl_3_ for compounds **4** and **5**: ^13^C chemical shift (δ_C_), ^1^H chemical shift (δ_H_), coupling constants (*J*, Hz).

| **4** | | | | | **5** | |
| --- | --- | --- | --- | --- | --- | --- |
| position | δ_C_, type | | δ_H_ (*J* in Hz) | | δ_C_, type | δ_H_ (*J* in Hz) |
| 1 | 148.3,CH | | 5.51 dd | | 43.8,CH | 2.73 t (6.7) |
|  |  | | (17.5,10.8) | |  |  |
| 2 | 113.3,CH_2_ | | 4.88 d (17.5) | | 41.8,CH_2_ | 2.35 m, |
|  |  | | 5.06 d (10.8) | |  | 2.74 d (19.8) |
| 3 | 174.7,C | |  | | 220.6,C |  |
| 4 | 36.6,CH_2_ | | 1.88 m | | 45.8,CH_2_ | 1.99 bd (13.3), |
|  |  | | 2.39 dd(15.2,2.5) | |  | 2.33 m |
| 5 | 42.8,CH | | 1.7 m | | 38.2,CH | 2.64 dqd (11.0. 8.4, 6.2) |
| 6 | 27.4,CH_2_ | | 1.22 m , | | 32.8,CH_2_ | 1.34 m, |
|  |  | | 1.61 m | |  | 2.17 dt (14.5, 8.4) |
| 7 | 31.5,CH_2_ | | 0.92 dd(12.5,3.4) | | 30.2,CH_2_ | 1.00 m, |
|  |  | | 1.66 m | |  | 1.80 m |
| 8 | 35.1,CH | | 1.27 m | | 41.9,CH | 1.27 m |
| 9 | 51.5,CH | | 0.84 m | | 52.3,CH | 1.33 m |
| 10 | 43.4,C | |  | | 80.2,C |  |
| 11 | 23.0,CH_2_ | | 1.24 m, | | 23.5,CH_2_ | 1.44 m, |
|  |  | | 1.33 m | |  | 1.64 m |
| 12 | 40.1,CH_2_ | | 1.1 m, | | 40.2,CH_2_ | 1.17 m, |
|  |  | | 1.9 m | |  | 2.00 m |
| 13 | 42.8,C | |  | | 43.2,C |  |
| 14 | 56.7,CH | | 0.99 m | | 57.1,CH | 1.15 m |
| 15 | 24.0,CH_2_ | | 1.12 m, | | 28.2,CH_2_ | 1.26 m, |
|  |  | | 1.31 m | |  | 1.83 m |
| 16 | 28.4,CH_2_ | | 1.23 m, | | 28.2,CH_2_ | 1.26 m, |
|  |  | | 1.82 m | |  | 1.83 m |
| 17 | 56.3,CH | | 1.06 m | | 56.6,CH | 1.11 m |
| 18 | 12.2,CH_3_ | | 0.63 s | | 12.4,CH_3_ | 0.72 s |
| 19 | 10.4,CH_3_ | | 0.82 s | | 19.1,CH_3_ | 1.0 s |
| 20 | 35.9,CH | | 1.35 m | | 35.6,CH | 1.38 m |
| 21 | 18.8,CH_3_ | | 0.87 d (6.5) | | 18.7,CH_3_ | 0.92 d (6.4) |
| 22 | 36.3,CH_2_ | | 0.98 m, | | 36.2,CH_2_ | 0.99 qnt (4.3), |
|  |  | | 1.31 m | |  | 1.33 m |
| 23 | 24.2,CH_2_ | | 1.04 m, | | 25.5,CH_2_ | 1.14 m, |
|  |  | | 1.55 m | |  | 1.67 m |
| 24 | 39.7,CH_2_ | | 1.08 m , | | 39.6,CH_2_ | 1.13 m, |
|  |  | | 1.12 m | |  | 1.13 m |
| 25 | 28.1,CH | | 1.50 n (6.6) | | 28.1,CH | 1.52 n (6.5) |
| 26 | 22.7,CH_3_ | | 0.84 d (6.4) | | 22.7,CH_3_ | 0.87 d (6.4) |
| 27 | 23.0,CH_3_ | | 0.85 d (6.4) | | 22.9,CH_3_ | 0.87 d (6.4) |
| OCH_3_ | 51.5 | | 3.62 s | | 47.6 | 3.00 s |
|  |  | |  | |  |  |
|  | |  | |  |  |  |

******

**Fig.S1.** ^1^H NMR spectrum of **4** (CDCl_3_, 600 MHz).

**Fig.S2.** ^13^C NMR spectrum of **4** (CDCl_3_, 150 MHz).

**Fig.S3.** ^1^H NMR spectrum of **5** (CDCl_3_, 600 MHz).

**Fig.S4.** ^13^C NMR spectrum of **5** (CDCl_3_, 150 MHz).

**Table S2**. NMR data recorded in CDCl_3_ for compound **6**: ^13^C chemical shift (δ_C_), ^1^H chemical shift (δ_H_), coupling constants (*J*, Hz).

| **6** | | |  |
| --- | --- | --- | --- |
| position | δ_C_, type | δ_H_ (*J* in Hz) |  |
| 1 | 45.8,CH | 3.02 q (7.8) |  |
| 2 | 44.3,CH_2_ | 2.38 dd (19.1, 5.0) |  |
|  |  | 2.41 dd (19.1, 8.4) |  |
| 3 | 220.0,C |  |  |
| 4 | 44.9,CH_2_ | 1.97 m, |  |
|  |  | 2.02 m |  |
| 5 | 38.9,CH | 2.51 m |  |
| 6 | 32.1,CH_2_ | 1.23 m, |  |
|  |  | 1.78 m |  |
| 7 | 31.9,CH_2_ | 1.17 m, |  |
|  |  | 1.77 m |  |
| 8 | 46,CH | 1.12 m |  |
| 9 | 51.6,CH | 1.66 dd (9.3, 3.6) |  |
| 10 | 154.8,C |  |  |
| 11 | 28.2,CH_2_ | 1.45 q (3.6) |  |
|  |  | 1.85 d (5.6) |  |
| 12 | 40.1,CH_2_ | 1.18 d (4.1), | |
|  |  | 2.00 dd (3.1, 1.6) |  |
| 13 | 43.0,C |  |  |
| 14 | 57.3,CH | 1.1 m |  |
| 15 | 24.7,CH_2_ | 1.09 m, |  |
|  |  | 1.62 m |  |
| 16 | 28.3,CH_2_ | 1.25 t (11.4), |  |
|  |  | 1.83 m |  |
| 17 | 56.3,CH | 1.11 m |  |
| 18 | 12.4,CH_3_ | 0.76 m |  |
| 19 | 107.9,CH_2_ | 4.61 s ,4.99 s |  |
| 20 | 35.9,CH | 1.37 m |  |
| 21 | 18.8,CH_3_ | 0.92 d (6.6) |  |
| 22 | 36.3,CH_2_ | 1.20 t (3.8), |  |
|  |  | 1.78 m |  |
| 23 | 25.8,CH_2_ | 1.06 m, |  |
|  |  | 1.84 d (4.8) |  |
| 24 | 23.9,CH_2_ | 1.14 m, |  |
|  |  | 1.33 m |  |
| 25 | 28.1,CH | 1.51 m |  |
| 26 | 22.7,CH_3_ | 0.86 d (6.4) |  |
| 27 | 22.9,CH_3_ | 0.85 d (6.4) |  |

**Fig.S5.** ^1^H NMR spectrum of **6** (CDCl_3_, 600 MHz).

**Fig.S6.** ^13^C NMR spectrum of **6** (CDCl_3_, 150 MHz).

**Table S3**. NMR data recorded in CDCl_3_ for compounds **10** and **11**: ^13^C chemical shift (δ_C_), ^1^H chemical shift (δ_H_), coupling constants (*J*, Hz).

|  | **10** | | **11** | |
| --- | --- | --- | --- | --- |
| position | δ_C_, type | δ_H_ (*J* in Hz) | δ_C_, type | δ_H_ (*J* in Hz) |
| 1 | 44.7,CH | 2.42 q (9.1) | 51.6,CH | 2.22 m |
| 2 | 37.8,CH_2_ | 1.77 m, | 38.4,CH_2_ | 1.72 m, |
|  |  | 2.18 dt (13.9, 8.3) |  | 2.22 m |
| 3 | 72.3,CH | 4.15 bqnt (8.1) | 72.2,CH | 4.14 m |
| 4 | 43.6,CH_2_ | 1.31, m, | 43.5,CH_2_ | 1.27 m, |
|  |  | 1.96 qnt (5.6) |  | 2.00 m |
| 5 | 39.4,CH | 2.05 m | 39.5,CH | 2.01 m |
| 6 | 31.1,CH_2_ | 1.35 m,1.84 m | 31.0,CH_2_ | 1.43 m, |
|  |  |  |  | 1.77 m |
| 7 | 32.7,CH_2_ | 1.76m, | 32.9,CH_2_ | 2.05 m, |
|  |  | 0.85 q (12.0) |  | 1.30 m |
| 8 | 40.3,CH | 1.48 m | 40.1,CH | 1.47 m |
| 9 | 52.4,CH | 1.29 m | 56.7,CH | 1.18 m |
| 10 | 80.7,C |  | 77.2,C |  |
| 11 | 23.2,CH_2_ | 1.43 m,1.63 m | 23.6,CH_2_ | 1.94 m, |
|  |  |  |  | 1.41 m |
| 12 | 40.3,CH_2_ | 1.12 td (12.8, 3.5) | 39.9,CH_2_ | 1.72 m, |
|  |  | 1.71 dt (12.8, 3.5) |  | 1.15 m |
| 13 | 41.0,C |  | 40.8,C |  |
| 14 | 57.0,CH | 1.20 m | 57.7,CH | 1.15 m |
| 15 | 32.9,CH_2_ | 1.31 m,2.05 m | 33.8,CH_2_ | 1.80 m, |
|  |  |  |  | 1.47 m, |
| 16 | 80.5,CH | 4.37 q (7.5) | 80.5,CH | 4.38 q (7.5) |
|  |  |  |  |  |
| 17 | 62.5,CH | 1.76 m | 62.4,CH | 1.76 m |
| 18 | 16.9,CH_3_ | 0.82 s | 16.7,CH_3_ | 0.81 s |
| 19 | 19.3,CH_3_ | 1.17 s | 19.9,CH_3_ | 1.25 s |
| 20 | 41.9,CH | 1.88 q (6.8) | 42.0,CH | 1.88 q (7.0) |
| 21 | 14.7,CH_3_ | 0.96 d (6.8) | 14.7,CH_3_ | 0.96 d (7.0) |
| 22 | 109.3,C |  | 109.3,C |  |
| 23 | 31.5,CH_2_ | 1.58 m | 31.5,CH_2_ | 1.67 m, |
|  |  | 1.66 dd (9.2,4.3) |  | 1.58 m |
| 24 | 28.9,CH_2_ | 1.43 m,1.60 m | 29.0,CH_2_ | 1.63 m, |
|  |  |  |  | 1.44 m |
| 25 | 30.4,CH | 1.62 m | 30.4,CH | 1.62 m |
| 26 | 17.3,CH_3_ | 0.78 d (6.2) | 17.3,CH_3_ | 0.78 d (6.2) |
| 27 | 67.0,CH_2_ | 3.37 t (10.8), | 67.0, CH_2_ | 3.36 t (10.9) |
|  |  | 3.46 bd (10.8) |  | 3.47 m |
| OCH_3_ | 47.9 | 3.02 s |  |  |

**Fig.S7.** ^1^H NMR spectrum of **10** (CDCl_3_, 600 MHz).

**Fig.S8.** ^13^C NMR spectrum of **10** (CDCl_3_, 150 MHz).

**Fig.S9.** ^1^H NMR spectrum of **11** (CDCl_3_, 400 MHz).

**Fig.S10.** ^13^C JMOD NMR spectrum of **11** (CDCl_3_, 101 MHz).

**Chart S1**. Chemical structures of compounds **4a** and **4b** considered for DFT calculations and differing for the configuration at C-10.

**Chart S2**. Chemical structures of compounds **5a** and **5b** considered for DFT calculations calculations and differing for the configuration at C-10.

**Chart S3**. Chemical structures of compounds **10a-d** considered for DFT calculations and differing for the configuration at C-3 and C-10.

**Chart S4**. Chemical structures of compounds **11a-d** considered for DFT calculations and differing for the configuration at C-3 and C-10.

**Table S4.** ^13^C/^1^H experimental and calculated NMR chemical shifts for **4a**-**b**, with ^a^|Δδ|(^13^C), ^b^|Δδ|(^1^H), and ^c^MAE values.

| **position** | **δ_exp_ (^13^C), ppm** | **δ_calc_ (^13^C), ppm** | | **\|Δδ\| (^13^C), ppm^a^** | | **position** | **δ_exp_ (^1^H), ppm** | **δ_calc_ (^1^H), ppm** | | **\|Δδ\| (^1^H), ppm^b^** | |
| --- | --- | --- | --- | --- | --- | --- | --- | --- | --- | --- | --- |
|  |  | **4a** | **4b** | **4a** | **4b** |  |  | **4a** | **4b** | **4a** | **4b** |
| OCH_3_ | 51.5 | 50.31 | 50.32 | 1.19 | 1.18 | OCH_3_ | 3.62 | 3.53 | 3.52 | 0.09 | 0.1 |
| 1 | 148.3 | 145.05 | 145.86 | 3.25 | 2.44 | 1 | 5.51 | 6.17 | 6.02 | 0.66 | 0.51 |
| 2 | 113.3 | 110.82 | 111.44 | 2.48 | 1.86 | 2 | 4.88 | 5.31 | 5.26 | 0.43 | 0.38 |
|  |  |  |  |  |  | 2 | 5.06 | 5.31 | 5.33 | 0.25 | 0.27 |
| 3 | 174.7 | 166.74 | 166.93 | IGNORED | IGNORED |  |  |  |  |  |  |
| 4 | 36.6 | 34.32 | 36.36 | 2.28 | 0.24 | 4 | 1.88 | 2.43 | 1.8 | 0.55 | 0.08 |
|  |  |  |  |  |  | 4 | 2.39 | 2.53 | 2.45 | 0.14 | 0.06 |
| 5 | 42.8 | 43.31 | 41.79 | 0.51 | 1.01 | 5 | 1.7 | 2.07 | 1.95 | 0.37 | 0.25 |
| 6 | 27.4 | 26.36 | 27.71 | 1.04 | 0.31 | 6 | 1.22 | 1.47 | 1.15 | 0.25 | 0.07 |
|  |  |  |  |  |  | 6 | 1.61 | 1.76 | 1.67 | 0.15 | 0.06 |
| 7 | 31.5 | 27.06 | 32.17 | 4.44 | 0.67 | 7 | 0.92 | 1.11 | 1.05 | 0.19 | 0.13 |
|  |  |  |  |  |  | 7 | 1.66 | 1.35 | 1.63 | 0.31 | 0.03 |
| 8 | 35.1 | 36.25 | 35.58 | 1.15 | 0.48 | 8 | 1.27 | 1.59 | 1.54 | 0.32 | 0.27 |
| 9 | 51.5 | 46.00 | 50.99 | 5.50 | 0.51 | 9 | 0.84 | 1.2 | 0.98 | 0.36 | 0.14 |
| 10 | 43.4 | 42.96 | 44.71 | 0.44 | 1.31 |  |  |  |  |  |  |
| 11 | 23.0 | 24.72 | 24.60 | 1.72 | 1.60 | 11 | 1.24 | 1.39 | 1.41 | 0.15 | 0.17 |
|  |  |  |  |  |  | 11 | 1.33 | 1.42 | 1.48 | 0.09 | 0.15 |
| 12 | 40.1 | 40.70 | 40.51 | 0.60 | 0.41 | 12 | 1.1 | 1.15 | 1.13 | 0.05 | 0.03 |
|  |  |  |  |  |  | 12 | 1.9 | 1.96 | 1.96 | 0.06 | 0.06 |
| 13 | 42.8 | 44.03 | 43.73 | 1.23 | 0.93 |  |  |  |  |  |  |
| 14 | 56.7 | 56.27 | 56.52 | 0.43 | 0.18 | 14 | 0.99 | 1.18 | 1.15 | 0.19 | 0.16 |
| 15 | 24.0 | 25.99 | 26.06 | 1.99 | 2.06 | 15 | 1.12 | 1.26 | 1.26 | 0.14 | 0.14 |
|  |  |  |  |  |  | 15 | 1.31 | 1.58 | 1.61 | 0.27 | 0.3 |
| 16 | 28.4 | 30.08 | 30.22 | 1.68 | 1.82 | 16 | 1.23 | 1.35 | 1.35 | 0.12 | 0.12 |
|  |  |  |  |  |  | 16 | 1.82 | 1.86 | 1.87 | 0.04 | 0.05 |
| 17 | 56.3 | 55.57 | 55.67 | 0.73 | 0.63 | 17 | 1.06 | 1.21 | 1.17 | 0.15 | 0.11 |
| 18 | 12.2 | 13.90 | 13.81 | 1.70 | 1.61 | 18 | 0.63 | 0.84 | 0.84 | 0.21 | 0.21 |
| 19 | 10.4 | 20.73 | 12.56 | 10.33 | 2.16 | 19 | 0.82 | 1.22 | 0.93 | 0.4 | 0.11 |
| 20 | 35.9 | 37.16 | 37.41 | 1.26 | 1.51 | 20 | 1.35 | 1.49 | 1.48 | 0.14 | 0.13 |
| 21 | 18.8 | 19.37 | 19.15 | 0.57 | 0.35 | 21 | 0.87 | 0.95 | 0.95 | 0.08 | 0.08 |
| 22 | 36.3 | 36.48 | 36.47 | 0.18 | 0.17 | 22 | 0.98 | 1.05 | 1.03 | 0.07 | 0.05 |
|  |  |  |  |  |  | 22 | 1.31 | 1.31 | 1.33 | 0 | 0.02 |
| 23 | 24.2 | 25.30 | 25.77 | 1.10 | 1.57 | 23 | 1.04 | 1.17 | 1.16 | 0.13 | 0.12 |
|  |  |  |  |  |  | 23 | 1.55 | 1.51 | 1.51 | 0.04 | 0.04 |
| 24 | 39.7 | 39.07 | 38.96 | 0.63 | 0.74 | 24 | 1.08 | 1.11 | 1.12 | 0.03 | 0.04 |
|  |  |  |  |  |  | 24 | 1.12 | 1.18 | 1.17 | 0.06 | 0.05 |
| 25 | 28.1 | 29.08 | 29.04 | 0.98 | 0.94 | 25 | 1.5 | 1.66 | 1.65 | 0.16 | 0.15 |
| 26 | 22.7 | 23.07 | 22.92 | 0.37 | 0.22 | 26 | 0.84 | 0.89 | 0.89 | 0.05 | 0.05 |
| 27 | 23.0 | 22.92 | 23.15 | 0.08 | 0.15 | 27 | 0.85 | 0.89 | 0.9 | 0.04 | 0.05 |
| **MAE^c^** |  |  |  | **1.77** | **1.00** |  |  |  |  | **0.19** | **0.13** |

**^a^ |Δδ|(^13^C)** = |δ_exp_ –δ_calc_| (^13^C), ppm: absolute differences for experimental versus calculated ^13^C NMR chemical shifts

**^b^ |Δδ|(^1^H)** = |δ_exp_ –δ_calc_| (^1^H), ppm: absolute differences for experimental versus calculated ^1^H NMR chemical shifts

**^c^ MAE** = Σ[|(δ_exp_ – δ_calcd_)|]/n, summation through n of the absolute error values (difference of the absolute values between corresponding experimental and ^13^C/^1^H chemical shifts), normalized to the number of the chemical shifts

**Table S5.** ^13^C/^1^H experimental and calculated NMR chemical shifts for **5a**-**b**, with ^a^|Δδ|(^13^C), ^b^|Δδ|(^1^H), and ^c^MAE values.

| **position** | **δ_exp_ (^13^C), ppm** | **δ_calc_ (^13^C), ppm** | | **\|Δδ\| (^13^C), ppm^a^** | | **position** | **δ_exp_ (^1^H), ppm** | **δ_calc_ (^1^H), ppm** | | **\|Δδ\| (^1^H), ppm^b^** | |
| --- | --- | --- | --- | --- | --- | --- | --- | --- | --- | --- | --- |
|  |  | **5a** | **5b** | **5a** | **5b** |  |  | **5a** | **5b** | **5a** | **5b** |
| 1 | 43.8 | 52.80 | 44.50 | 9.00 | 0.70 | 1 | 2.73 | 2.14 | 2.53 | 0.59 | 0.20 |
| 2 | 41.8 | 39.20 | 41.20 | 2.60 | 0.60 | 2 | 2.35 | 1.94 | 2.22 | 0.41 | 0.13 |
|  |  |  |  |  |  | 2 | 2.74 | 2.87 | 2.85 | 0.13 | 0.11 |
| 3 | 220.6 | 208.70 | 209.90 | IGNORED | IGNORED |  |  |  |  |  |  |
| 4 | 45.8 | 48.40 | 45.90 | 2.60 | 0.10 | 4 | 1.99 | 1.86 | 1.93 | 0.13 | 0.06 |
|  |  |  |  |  |  | 4 | 2.33 | 2.31 | 2.08 | 0.02 | 0.25 |
| 5 | 38.2 | 40.60 | 39.10 | 2.40 | 0.90 | 5 | 2.64 | 2.20 | 2.47 | 0.44 | 0.17 |
| 6 | 32.8 | 30.60 | 34.10 | 2.20 | 1.30 | 6 | 1.34 | 1.07 | 1.33 | 0.27 | 0.01 |
|  |  |  |  |  |  | 6 | 2.17 | 2.43 | 2.06 | 0.26 | 0.11 |
| 7 | 30.2 | 38.70 | 31.00 | 8.50 | 0.80 | 7 | 1.00 | 0.97 | 1.06 | 0.03 | 0.06 |
|  |  |  |  |  |  | 7 | 1.80 | 1.90 | 1.63 | 0.10 | 0.17 |
| 8 | 41.9 | 37.40 | 42.50 | 4.50 | 0.60 | 8 | 1.27 | 2.00 | 1.47 | 0.73 | 0.20 |
| 9 | 52.3 | 48.00 | 51.90 | 4.30 | 0.40 | 9 | 1.33 | 1.50 | 1.36 | 0.17 | 0.03 |
| 10 | 80.2 | 77.40 | 78.40 | 2.80 | 1.80 |  |  |  |  |  |  |
| 11 | 23.5 | 29.90 | 24.80 | 6.40 | 1.30 | 11 | 1.44 | 1.60 | 1.58 | 0.16 | 0.14 |
|  |  |  |  |  |  | 11 | 1.64 | 1.91 | 1.80 | 0.27 | 0.16 |
| 12 | 40.2 | 41.10 | 40.50 | 0.90 | 0.30 | 12 | 1.17 | 1.11 | 1.15 | 0.06 | 0.02 |
|  |  |  |  |  |  | 12 | 2.00 | 2.04 | 2.02 | 0.04 | 0.02 |
| 13 | 43.2 | 43.00 | 44.20 | 0.20 | 1.00 |  |  |  |  |  |  |
| 14 | 57.1 | 58.20 | 57.00 | 1.10 | 0.10 | 14 | 1.15 | 1.17 | 1.25 | 0.02 | 0.10 |
| 15 | 28.2 | 27.10 | 27.00 | 1.10 | 1.20 | 15 | 1.26 | 1.29 | 1.30 | 0.03 | 0.04 |
|  |  |  |  |  |  | 15 | 1.83 | 1.69 | 1.61 | 0.14 | 0.22 |
| 16 | 28.2 | 29.90 | 29.60 | 1.70 | 1.40 | 16 | 1.26 | 1.37 | 1.38 | 0.11 | 0.12 |
|  |  |  |  |  |  | 16 | 1.83 | 1.87 | 1.86 | 0.04 | 0.03 |
| 17 | 56.6 | 55.50 | 55.90 | 1.10 | 0.70 | 17 | 1.11 | 1.20 | 1.24 | 0.09 | 0.13 |
| 18 | 12.4 | 14.10 | 14.00 | 1.70 | 1.60 | 18 | 0.72 | 0.90 | 0.89 | 0.18 | 0.17 |
| 19 | 19.1 | 23.90 | 20.70 | 4.80 | 1.60 | 19 | 1.00 | 1.15 | 0.99 | 0.15 | 0.01 |
| 20 | 35.6 | 37.40 | 36.90 | 1.80 | 1.30 | 20 | 1.38 | 1.48 | 1.52 | 0.10 | 0.14 |
| 21 | 18.7 | 19.50 | 19.40 | 0.80 | 0.70 | 21 | 0.92 | 0.97 | 0.98 | 0.05 | 0.06 |
| 22 | 36.2 | 35.60 | 36.00 | 0.60 | 0.20 | 22 | 0.99 | 1.04 | 1.04 | 0.05 | 0.05 |
|  |  |  |  |  |  | 22 | 1.33 | 1.35 | 1.35 | 0.02 | 0.02 |
| 23 | 25.5 | 25.20 | 25.40 | 0.30 | 0.10 | 23 | 1.14 | 1.24 | 1.29 | 0.10 | 0.15 |
|  |  |  |  |  |  | 23 | 1.67 | 1.50 | 1.45 | 0.17 | 0.22 |
| 24 | 39.6 | 38.60 | 38.80 | 1.00 | 0.80 | 24 | 1.13 | 1.15 | 1.13 | 0.02 | 0.00 |
|  |  |  |  |  |  | 24 | 1.13 | 1.20 | 1.19 | 0.07 | 0.06 |
| 25 | 28.1 | 28.10 | 28.80 | 0.00 | 0.70 | 25 | 1.52 | 1.70 | 1.66 | 0.18 | 0.14 |
| 26 | 22.7 | 22.30 | 22.40 | 0.40 | 0.30 | 26 | 0.87 | 0.88 | 0.89 | 0.01 | 0.02 |
| 27 | 22.9 | 23.40 | 23.60 | 0.50 | 0.70 | 27 | 0.87 | 0.89 | 0.89 | 0.02 | 0.02 |
| OCH3 | 47.6 | 51.30 | 46.60 | 3.70 | 1.00 | OCH3 | 3.00 | 3.46 | 2.99 | 0.46 | 0.01 |
| **MAE^c^** |  |  |  | **2.48** | **0.82** |  |  |  |  | **0.16** | **0.10** |

**^a^ |Δδ|(^13^C)** = |δ_exp_ –δ_calc_| (^13^C), ppm: absolute differences for experimental versus calculated ^13^C NMR chemical shifts

**^b^ |Δδ|(^1^H)** = |δ_exp_ –δ_calc_| (^1^H), ppm: absolute differences for experimental versus calculated ^1^H NMR chemical shifts

**^c^ MAE** = Σ[|(δ_exp_ – δ_calcd_)|]/n, summation through n of the absolute error values (difference of the absolute values between corresponding experimental and ^13^C/^1^H chemical shifts), normalized to the number of the chemical shifts

**Table S6.** ^13^C/^1^H experimental and calculated NMR chemical shifts for **10a**-**d**, with ^a^|Δδ|(^13^C), ^b^|Δδ|(^1^H), and ^c^MAE values.

| **position** | **δ_exp_ (^13^C), ppm** | **δ_calc_ (^13^C), ppm** | | | | **\|Δδ\| (^13^C), ppm^a^** | | | | **position** | **δ_exp_ (^1^H), ppm** | **δ_calc_ (^1^H), ppm** | | | | **\|Δδ\| (^1^H), ppm^b^** | | | | | | | |
| --- | --- | --- | --- | --- | --- | --- | --- | --- | --- | --- | --- | --- | --- | --- | --- | --- | --- | --- | --- | --- | --- | --- | --- |
|  |  | **10a** | **10b** | **10c** | **10d** | **10a** | **10b** | **10c** | **10d** |  |  | **10a** | **10b** | **10c** | **10d** | **10a** | | **10b** | | **10c** | | **10d** | |
| 1 | 44.7 | 56.10 | 44.50 | 53.10 | 45.10 | 11.40 | 0.20 | 8.40 | 0.40 | 1 | 2.42 | 1.83 | 2.33 | 1.97 | 2.58 | 0.59 | | 0.09 | | 0.45 | | 0.16 | |
| 2 | 37.8 | 40.40 | 38.40 | 39.90 | 40.20 | 2.60 | 0.60 | 2.10 | 2.40 | 2 | 1.77 | 1.41 | 1.63 | 1.68 | 1.54 | 0.36 | | 0.14 | | 0.09 | | 0.23 | |
|  |  |  |  |  |  |  |  |  |  | 2 | 2.18 | 2.05 | 2.23 | 2.04 | 2.37 | 0.13 | | 0.05 | | 0.14 | | 0.19 | |
| 3 | 72.3 | 70.90 | 71.70 | 72.30 | 72.00 | 1.40 | 0.60 | 0.00 | 0.30 | 3 | 4.15 | 3.98 | 4.04 | 4.07 | 4.25 | 0.17 | | 0.11 | | 0.08 | | 0.10 | |
| 4 | 43.6 | 43.90 | 44.00 | 44.40 | 42.90 | 0.30 | 0.40 | 0.80 | 0.70 | 4 | 1.31 | 1.27 | 1.03 | 1.48 | 1.28 | 0.04 | | 0.28 | | 0.17 | | 0.03 | |
|  |  |  |  |  |  |  |  |  |  | 4 | 1.96 | 2.13 | 1.75 | 1.77 | 1.72 | 0.17 | | 0.21 | | 0.19 | | 0.24 | |
| 5 | 39.4 | 44.00 | 39.80 | 41.40 | 40.20 | 4.60 | 0.40 | 2.00 | 0.80 | 5 | 2.05 | 2.01 | 1.94 | 2.07 | 2.73 | 0.04 | | 0.11 | | 0.02 | | 0.68 | |
| 6 | 31.1 | 31.90 | 32.30 | 31.30 | 31.90 | 0.80 | 1.20 | 0.20 | 0.80 | 6 | 1.35 | 1.78 | 1.40 | 1.34 | 1.36 | 0.43 | | 0.05 | | 0.01 | | 0.01 | |
|  |  |  |  |  |  |  |  |  |  | 6 | 1.84 | 2.00 | 1.75 | 2.33 | 1.84 | 0.16 | | 0.09 | | 0.49 | | 0.00 | |
| 7 | 32.7 | 33.50 | 32.30 | 38.20 | 32.50 | 0.80 | 0.40 | 5.50 | 0.20 | 7 | 0.85 | 1.03 | 0.91 | 0.88 | 1.02 | 0.18 | | 0.06 | | 0.03 | | 0.17 | |
|  |  |  |  |  |  |  |  |  |  | 7 | 1.76 | 1.96 | 1.68 | 1.97 | 1.72 | 0.20 | | 0.08 | | 0.21 | | 0.04 | |
| 8 | 40.3 | 40.70 | 41.70 | 38.20 | 41.80 | 0.40 | 1.40 | 2.10 | 1.50 | 8 | 1.48 | 2.02 | 1.65 | 2.10 | 1.68 | 0.54 | | 0.17 | | 0.62 | | 0.20 | |
| 9 | 52.4 | 55.00 | 52.40 | 51.20 | 52.30 | 2.60 | 0.00 | 1.20 | 0.10 | 9 | 1.29 | 1.15 | 1.26 | 1.23 | 1.30 | 0.14 | | 0.03 | | 0.06 | | 0.01 | |
| 10 | 80.7 | 80.40 | 78.80 | 79.30 | 78.80 | 0.30 | 1.90 | 1.40 | 1.90 |  |  |  |  |  |  |  | |  | |  | |  | |
| 11 | 23.2 | 29.20 | 24.80 | 30.10 | 24.70 | 6.00 | 1.60 | 6.90 | 1.50 | 11 | 1.43 | 1.66 | 1.46 | 1.59 | 1.48 | 0.23 | | 0.03 | | 0.16 | | 0.05 | |
|  |  |  |  |  |  |  |  |  |  | 11 | 1.63 | 1.78 | 1.74 | 1.83 | 1.73 | 0.15 | | 0.11 | | 0.20 | | 0.10 | |
| 12 | 40.3 | 41.10 | 40.60 | 41.10 | 40.50 | 0.80 | 0.30 | 0.80 | 0.20 | 12 | 1.12 | 1.00 | 1.06 | 1.02 | 1.07 | 0.12 | | 0.06 | | 0.10 | | 0.05 | |
|  |  |  |  |  |  |  |  |  |  | 12 | 1.71 | 1.73 | 1.70 | 1.71 | 1.70 | 0.02 | | 0.01 | | 0.00 | | 0.01 | |
| 13 | 41.1 | 41.40 | 42.40 | 41.10 | 42.50 | 0.30 | 1.30 | 0.00 | 1.40 |  |  |  |  |  |  |  | |  | |  | |  | |
| 14 | 57.0 | 56.80 | 57.10 | 57.70 | 57.10 | 0.20 | 0.10 | 0.70 | 0.10 | 14 | 1.20 | 1.26 | 1.23 | 1.22 | 1.26 | 0.06 | | 0.03 | | 0.02 | | 0.06 | |
| 15 | 32.9 | 33.80 | 33.50 | 34.00 | 33.50 | 0.90 | 0.60 | 1.10 | 0.60 | 15 | 1.31 | 1.57 | 1.52 | 1.56 | 1.53 | 0.26 | | 0.21 | | 0.25 | | 0.22 | |
|  |  |  |  |  |  |  |  |  |  | 15 | 2.05 | 2.10 | 1.96 | 2.09 | 1.99 | 0.05 | | 0.09 | | 0.04 | | 0.06 | |
| 16 | 80.5 | 79.70 | 79.80 | 79.90 | 79.80 | 0.80 | 0.70 | 0.60 | 0.70 | 16 | 4.37 | 4.59 | 4.55 | 4.59 | 4.56 | 0.22 | | 0.18 | | 0.22 | | 0.19 | |
| 17 | 62.5 | 62.90 | 62.90 | 62.60 | 63.10 | 0.40 | 0.40 | 0.10 | 0.60 | 17 | 1.76 | 1.67 | 1.67 | 1.68 | 1.68 | 0.09 | | 0.09 | | 0.08 | | 0.08 | |
| 18 | 16.9 | 18.20 | 18.30 | 18.20 | 18.20 | 1.30 | 1.40 | 1.30 | 1.30 | 18 | 0.82 | 0.98 | 0.96 | 0.99 | 0.97 | 0.16 | | 0.14 | | 0.17 | | 0.15 | |
| 19 | 19.3 | 22.50 | 20.50 | 23.30 | 19.90 | 3.20 | 1.20 | 4.00 | 0.60 | 19 | 1.17 | 1.30 | 1.10 | 1.12 | 1.02 | 0.13 | | 0.07 | | 0.05 | | 0.15 | |
| 20 | 41.9 | 43.60 | 43.60 | 43.60 | 43.50 | 1.70 | 1.70 | 1.70 | 1.60 | 20 | 1.88 | 1.90 | 1.90 | 1.89 | 1.92 | 0.02 | | 0.02 | | 0.01 | | 0.04 | |
| 21 | 14.7 | 16.60 | 16.60 | 16.60 | 16.60 | 1.90 | 1.90 | 1.90 | 1.90 | 21 | 0.96 | 0.97 | 0.97 | 0.97 | 0.98 | 0.01 | | 0.01 | | 0.01 | | 0.02 | |
| 22 | 109.3 | 106.70 | 106.90 | 107.00 | 106.80 | 2.60 | 2.40 | 2.30 | 2.50 |  |  |  |  |  |  |  | |  | |  | |  | |
| 23 | 31.5 | 32.30 | 32.20 | 32.20 | 32.20 | 0.80 | 0.70 | 0.70 | 0.70 | 23 | 1.58 | 1.42 | 1.42 | 1.44 | 1.42 | 0.16 | | 0.16 | | 0.14 | | 0.16 | |
|  |  |  |  |  |  |  |  |  |  | 23 | 1.66 | 1.55 | 1.57 | 1.54 | 1.56 | 0.11 | | 0.09 | | 0.12 | | 0.10 | |
| 24 | 28.9 | 29.40 | 29.60 | 29.40 | 29.60 | 0.50 | 0.70 | 0.50 | 0.70 | 24 | 1.43 | 1.40 | 1.41 | 1.38 | 1.41 | 0.03 | | 0.02 | | 0.05 | | 0.02 | |
|  |  |  |  |  |  |  |  |  |  | 24 | 1.60 | 1.55 | 1.59 | 1.58 | 1.60 | 0.05 | | 0.01 | | 0.02 | | 0.00 | |
| 25 | 30.4 | 30.30 | 30.50 | 30.30 | 30.50 | 0.10 | 0.10 | 0.10 | 0.10 | 25 | 1.62 | 1.59 | 1.60 | 1.55 | 1.60 | 0.03 | | 0.02 | | 0.07 | | 0.02 | |
| 26 | 17.3 | 18.50 | 18.50 | 18.60 | 18.50 | 1.20 | 1.20 | 1.30 | 1.20 | 26 | 0.78 | 0.70 | 0.69 | 0.69 | 0.69 | 0.08 | | 0.09 | | 0.09 | | 0.09 | |
| 27 | 67.0 | 65.20 | 65.00 | 65.20 | 65.00 | 1.80 | 2.00 | 1.80 | 2.00 | 27 | 3.37 | 3.40 | 3.38 | 3.40 | 3.38 | 0.03 | | 0.01 | | 0.03 | | 0.01 | |
|  |  |  |  |  |  |  |  |  |  | 27 | 3.46 | 3.56 | 3.56 | 3.58 | 3.57 | 0.10 | | 0.10 | | 0.12 | | 0.11 | |
| OCH_3_ | 47.9 | 50.20 | 47.00 | 51.10 | 46.90 | 2.30 | 0.90 | 3.20 | 1.00 | OCH_3_ | 3.02 | 3.44 | 3.00 | 3.43 | 3.03 | 0.42 | 0.02 | | 0.41 | | 0.01 | |  |
|  |  |  |  |  |  |  |  |  |  | OH | ND^d^ | 3.11 | 0.76 | 0.11 | 0.28 | NC^e^ | | NC^e^ | | NC^e^ | | NC^e^ | |
| **MAE^c^** |  |  |  |  |  | **1.86** | **0.94** | **1.88** | **0.99** | **MAE^c^** |  |  |  |  |  | **0.16** | | **0.09** | | **0.14** | | **0.11** | |

**^a^ |Δδ|(^13^C)** = |δ_exp_ –δ_calc_| (^13^C), ppm: absolute differences for experimental versus calculated ^13^C NMR chemical shifts

**^b^ |Δδ|(^1^H)** = |δ_exp_ –δ_calc_| (^1^H), ppm: absolute differences for experimental versus calculated ^1^H NMR chemical shifts

**^c^ MAE** = Σ[|(δ_exp_ – δ_calcd_)|]/n, summation through n of the absolute error values (difference of the absolute values between corresponding experimental and ^13^C/^1^H chemical shifts), normalized to the number of the chemical shifts

**^d^** **ND**: not determined

**^e^ NC**: not computed

**Table S7.** ^13^C/^1^H experimental and calculated NMR chemical shifts for **11a**-**d**, with ^a^|Δδ|(^13^C), ^b^|Δδ|(^1^H), and ^c^MAE values.

| **position** | **δ_exp_ (^13^C), ppm** | **δ_calc_ (^13^C), ppm** | | | | **\|Δδ\| (^13^C), ppm^a^** | | | | **position** | **δ_exp_ (^1^H), ppm** | **δ_calc_ (^1^H), ppm** | | | | **\|Δδ\| (^1^H), ppm^b^** | | | |
| --- | --- | --- | --- | --- | --- | --- | --- | --- | --- | --- | --- | --- | --- | --- | --- | --- | --- | --- | --- |
|  |  | **11a** | **11b** | **11c** | **11d** | **11a** | **11b** | **11c** | **11d** |  |  | **11a** | **11b** | **11c** | **11d** | **11a** | **11b** | **11c** | **11d** |
| 1 | 51.6 | 53.50 | 52.50 | 51.50 | 53.50 | 1.90 | 0.90 | 0.10 | 1.90 | 1 | 2.22 | 2.00 | 2.22 | 2.25 | 2.05 | 0.22 | 0.00 | 0.03 | 0.17 |
| 2 | 38.4 | 38.70 | 37.40 | 37.10 | 41.90 | 0.30 | 1.00 | 1.30 | 3.50 | 2 | 2.22 | 1.37 | 2.05 | 2.29 | 1.46 | 0.85 | 0.17 | 0.07 | 0.76 |
|  |  |  |  |  |  |  |  |  |  | 2 | 1.72 | 2.39 | 1.66 | 1.61 | 2.40 | 0.67 | 0.06 | 0.11 | 0.68 |
| 3 | 72.2 | 73.00 | 70.90 | 71.10 | 71.80 | 0.80 | 1.30 | 1.10 | 0.40 | 3 | 4.14 | 4.29 | 4.15 | 4.36 | 4.26 | 0.15 | 0.01 | 0.22 | 0.12 |
| 4 | 43.5 | 45.40 | 44.30 | 45.10 | 42.20 | 1.90 | 0.80 | 1.60 | 1.30 | 4 | 2 | 2.20 | 1.51 | 1.55 | 1.77 | 0.20 | 0.49 | 0.45 | 0.23 |
|  |  |  |  |  |  |  |  |  |  | 4 | 1.27 | 1.22 | 1.38 | 1.80 | 1.24 | 0.05 | 0.11 | 0.53 | 0.03 |
| 5 | 39.5 | 45.20 | 40.20 | 42.90 | 40.50 | 5.70 | 0.70 | 3.40 | 1.00 | 5 | 2.01 | 2.12 | 1.95 | 2.03 | 2.72 | 0.11 | 0.06 | 0.02 | 0.71 |
| 6 | 31 | 34.40 | 32.30 | 32.00 | 31.60 | 3.40 | 1.30 | 1.00 | 0.60 | 6 | 1.77 | 1.89 | 1.61 | 1.27 | 1.83 | 0.12 | 0.16 | 0.50 | 0.06 |
|  |  |  |  |  |  |  |  |  |  | 6 | 1.43 | 2.03 | 1.57 | 1.96 | 1.40 | 0.60 | 0.14 | 0.53 | 0.03 |
| 7 | 32.9 | 32.90 | 34.10 | 38.20 | 33.00 | 0.00 | 1.20 | 5.30 | 0.10 | 7 | 1.3 | 1.74 | 1.75 | 1.87 | 1.76 | 0.44 | 0.45 | 0.57 | 0.46 |
|  |  |  |  |  |  |  |  |  |  | 7 | 2.05 | 1.04 | 0.92 | 0.91 | 1.07 | 1.01 | 1.13 | 1.14 | 0.98 |
| 8 | 40.1 | 41.50 | 40.60 | 37.50 | 42.00 | 1.40 | 0.50 | 2.60 | 1.90 | 8 | 1.47 | 2.14 | 1.67 | 2.10 | 1.68 | 0.67 | 0.20 | 0.63 | 0.21 |
| 9 | 56.7 | 56.30 | 56.70 | 49.20 | 61.00 | 0.40 | 0.00 | 7.50 | 4.30 | 9 | 1.18 | 0.91 | 1.13 | 1.30 | 0.69 | 0.27 | 0.05 | 0.12 | 0.49 |
| 10 | 77.2 | 74.10 | 74.90 | 74.90 | 75.10 | 3.10 | 2.30 | 2.30 | 2.10 |  |  |  |  |  |  |  |  |  |  |
| 11 | 23.6 | 27.00 | 24.60 | 27.10 | 25.30 | 3.40 | 1.00 | 3.50 | 1.70 | 11 | 1.94 | 1.65 | 2.31 | 1.52 | 2.17 | 0.29 | 0.37 | 0.42 | 0.23 |
|  |  |  |  |  |  |  |  |  |  | 11 | 1.41 | 1.77 | 1.45 | 1.61 | 1.50 | 0.36 | 0.04 | 0.20 | 0.09 |
| 12 | 39.9 | 40.90 | 40.20 | 40.60 | 40.60 | 1.00 | 0.30 | 0.70 | 0.70 | 12 | 1.72 | 1.72 | 1.68 | 1.72 | 1.71 | 0.00 | 0.04 | 0.00 | 0.01 |
|  |  |  |  |  |  |  |  |  |  | 12 | 1.15 | 1.05 | 1.05 | 1.08 | 1.01 | 0.10 | 0.10 | 0.07 | 0.14 |
| 13 | 40.8 | 41.60 | 42.20 | 41.10 | 42.40 | 0.80 | 1.40 | 0.30 | 1.60 |  |  |  |  |  |  |  |  |  |  |
| 14 | 57.7 | 56.30 | 56.70 | 57.30 | 57.00 | 1.40 | 1.00 | 0.40 | 0.70 | 14 | 1.15 | 1.28 | 1.20 | 1.23 | 1.23 | 0.13 | 0.05 | 0.08 | 0.08 |
| 15 | 33.8 | 33.90 | 33.70 | 33.90 | 33.50 | 0.10 | 0.10 | 0.10 | 0.30 | 15 | 1.8 | 2.00 | 1.98 | 2.00 | 1.99 | 0.20 | 0.18 | 0.20 | 0.19 |
|  |  |  |  |  |  |  |  |  |  | 15 | 1.47 | 1.55 | 1.50 | 1.51 | 1.53 | 0.08 | 0.03 | 0.04 | 0.06 |
| 16 | 80.5 | 79.90 | 79.90 | 80.00 | 79.90 | 0.60 | 0.60 | 0.50 | 0.60 | 16 | 4.38 | 4.54 | 4.56 | 4.58 | 4.57 | 0.16 | 0.18 | 0.20 | 0.19 |
| 17 | 62.4 | 63.10 | 63.10 | 62.60 | 63.00 | 0.70 | 0.70 | 0.20 | 0.60 | 17 | 1.76 | 1.67 | 1.66 | 1.67 | 1.67 | 0.09 | 0.10 | 0.09 | 0.09 |
| 18 | 16.7 | 17.80 | 17.90 | 17.80 | 18.20 | 1.10 | 1.20 | 1.10 | 1.50 | 18 | 0.81 | 0.97 | 0.95 | 0.95 | 0.96 | 0.16 | 0.14 | 0.14 | 0.15 |
| 19 | 19.9 | 28.40 | 20.60 | 30.30 | 17.90 | 8.50 | 0.70 | 10.40 | 2.00 | 19 | 1.25 | 1.17 | 1.22 | 0.96 | 1.08 | 0.08 | 0.03 | 0.29 | 0.17 |
| 20 | 42 | 43.70 | 43.60 | 43.50 | 43.50 | 1.70 | 1.60 | 1.50 | 1.50 | 20 | 1.88 | 1.93 | 1.93 | 1.89 | 1.92 | 0.05 | 0.05 | 0.01 | 0.04 |
| 21 | 14.7 | 16.60 | 16.70 | 16.60 | 16.60 | 1.90 | 2.00 | 1.90 | 1.90 | 21 | 0.96 | 0.97 | 0.98 | 0.97 | 0.97 | 0.01 | 0.02 | 0.01 | 0.01 |
| 22 | 109.3 | 106.90 | 106.70 | 106.90 | 106.70 | 2.40 | 2.60 | 2.40 | 2.60 |  |  |  |  |  |  |  |  |  |  |
| 23 | 31.5 | 32.10 | 32.30 | 32.30 | 32.20 | 0.60 | 0.80 | 0.80 | 0.70 | 23 | 1.67 | 1.56 | 1.55 | 1.55 | 1.55 | 0.11 | 0.12 | 0.12 | 0.12 |
|  |  |  |  |  |  |  |  |  |  | 23 | 1.58 | 1.41 | 1.40 | 1.41 | 1.40 | 0.17 | 0.18 | 0.17 | 0.18 |
| 24 | 29 | 29.60 | 29.50 | 29.40 | 29.50 | 0.60 | 0.50 | 0.40 | 0.50 | 24 | 1.63 | 1.59 | 1.57 | 1.54 | 1.57 | 0.04 | 0.06 | 0.09 | 0.06 |
|  |  |  |  |  |  |  |  |  |  | 24 | 1.44 | 1.42 | 1.41 | 1.38 | 1.39 | 0.02 | 0.03 | 0.06 | 0.05 |
| 25 | 30.4 | 30.40 | 30.40 | 30.50 | 30.50 | 0.00 | 0.00 | 0.10 | 0.10 | 25 | 1.62 | 1.61 | 1.61 | 1.58 | 1.60 | 0.01 | 0.01 | 0.04 | 0.02 |
| 26 | 17.3 | 18.50 | 18.50 | 18.40 | 18.50 | 1.20 | 1.20 | 1.10 | 1.20 | 26 | 0.78 | 0.70 | 0.69 | 0.69 | 0.69 | 0.08 | 0.09 | 0.09 | 0.09 |
| 27 | 67 | 65.00 | 65.00 | 65.30 | 65.10 | 2.00 | 2.00 | 1.70 | 1.90 | 27 | 3.47 | 3.56 | 3.56 | 3.55 | 3.55 | 0.09 | 0.09 | 0.08 | 0.08 |
|  |  |  |  |  |  |  |  |  |  | 27 | 3.36 | 3.39 | 3.38 | 3.39 | 3.38 | 0.03 | 0.02 | 0.03 | 0.02 |
| **MAE^c^** |  |  |  |  |  | **1.74** | **1.03** | **1.97** | **1.38** |  |  |  |  |  |  | **0.22** | **0.15** | **0.22** | **0.21** |

**^a^ |Δδ|(^13^C)** = |δ_exp_ –δ_calc_| (^13^C), ppm: absolute differences for experimental versus calculated ^13^C NMR chemical shifts

**^b^ |Δδ|(^1^H)** = |δ_exp_ –δ_calc_| (^1^H), ppm: absolute differences for experimental versus calculated ^1^H NMR chemical shifts

**^c^ MAE** = Σ[|(δ_exp_ – δ_calcd_)|]/n, summation through n of the absolute error values (difference of the absolute values between corresponding experimental and ^13^C/^1^H chemical shifts), normalized to the number of the chemical shifts

**Table S8.** DP4+ probabilities obtained for compounds **4a**-**4b**, **5a**-**5b**, **10a**-**d**, **11a**-**d**.

|  | **DP4+ (^13^C DATA)** | **DP4+ (^1^H DATA)** | **DP4+ (all DATA)** |
| --- | --- | --- | --- |
| **4a** | 0.00% | 0.00% | 0.00% |
| **4b** | 100.00% | 100.00% | 100.00% |
|  |  |  |  |
| **5a** | 0.00% | 0.00% | 0.00% |
| **5b** | 100.00% | 100.00% | 100.00% |
|  |  |  |  |
| **10a** | 0.00% | 0.00% | 0.00% |
| **10b** | 100.00% | 96.12% | 100.00% |
| **10c** | 0.00% | 0.00% | 0.00% |
| **10d** | 0.00% | 3.88% | 0.00% |
|  |  |  |  |
| **11a** | 0.00% | 0.00% | 0.00% |
| **11b** | 100.00% | 100.00% | 100.00% |
| **11c** | 0.00% | 0.00% | 0.00% |
| **11d** | 0.01% | 0.00% | 0.00% |

References:

[1] Maestro 12.7; Schrödinger, LLC, New York, NY, **2021**.

[2] MacroModel 13.1; Schrödinger, LLC, New York, NY, **2021**.

[3] M. J. Frisch, G. W. Trucks, H. B. Schlegel, G. E. Scuseria, M. A. Robb, J. R. Cheeseman, G. Scalmani, V. Barone, B. Mennucci, G. A. Petersson, H. Nakatsuji, M. Caricato, X. Li, H. P. Hratchian, A. F. Izmaylov, J. Bloino, G. Zheng, J. L. Sonnenberg, M. Hada, M. Ehara, K. Toyota, R. Fukuda, J. Hasegawa, M. Ishida, T. Nakajima, Y. Honda, O. Kitao, H. Nakai, T. Vreven, J. A. Montgomery, Jr., J. E. Peralta, F. Ogliaro, M. Bearpark, J. J. Heyd, E. Brothers, K. N. Kudin, V. N. Staroverov, R. Kobayashi, J. Normand, K. Raghavachari, A. Rendell, J. C. Burant, S. S. Iyengar, J. Tomasi, M. Cossi, N. Rega, J. M. Millam, M. Klene, J. E. Knox, J. B. Cross, V. Bakken, C. Adamo, J. Jaramillo, R. Gomperts, R. E. Stratmann, O. Yazyev, A. J. Austin, R. Cammi, C. Pomelli, J. W. Ochterski, R. L. Martin, K. Morokuma, V. G. Zakrzewski, G. A. Voth, P. Salvador, J. J. Dannenberg, S. Dapprich, A. D. Daniels, Ö. Farkas, J. B. Foresman, J. V. Ortiz, J. Cioslowski, and D. J. Fox, *Gaussian 09*, Gaussian, Inc., Wallingford CT, **2009**.

[4] P. Cimino, L. Gomez-Paloma, D. Duca, R. Riccio, G. Bifulco, *Magn. Reson. Chem.* **2004**, *42*, 26–33.

[5] N. Grimblat, M. M. Zanardi, A. M. Sarotti, *J. Org. Chem.* **2015**, *80*, 12526–12534.
